# Exploring customer retention dynamics: A comparative investigation of factors affecting customer retention in the banking sector using mediation-moderation approach

**DOI:** 10.1016/j.heliyon.2024.e36919

**Published:** 2024-08-29

**Authors:** Chai Zhengmeng, Muhammad Malik, Muttahir Hussain, Salamat Hussain

**Affiliations:** a Faculty of Management and Economics, Kunming University of Science and Technology, Yunnan, China; b School of Management, Guangzhou University, China

**Keywords:** Customer trust, Customer satisfaction, Customer retention behavior, Cultural difference, Financial technology, Service quality

## Abstract

The purpose of this research is to explore the impact of service quality, customer trust, and cultural disparities on customer satisfaction within the banking sector. Additionally, the study examined the mediating effect of customer satisfaction and the moderating effect of financial technology. Data for this investigation were gathered from customers utilizing services offered by public banks in Pakistan and China. The analysis was conducted using the Smart-PLS software, with 281 samples from Pakistan and 312 samples from China being included in the study. Results show that service quality, customer trust, and cultural differences positively affect customer satisfaction in the context of Pakistan and China. Results also reveal that service quality and customer trust have a positive effect on customer retention behavior in China, but customer trust has an insignificant impact on customer retention behavior in Pakistan. Analysis specifies that customer satisfaction positively mediated the relationship between service quality, customer trust, cultural differences, and customer retention behavior in Pakistan and China. More importantly, financial technology moderated the relationship between customer satisfaction and customer retention behavior, but it was insignificant in the context of China and Pakistan. The study seeks to provide significant insights for strategic decision-making and improve customer relationship management techniques in the dynamic field of financial services by including mediating and moderating elements.

## 1 Introduction

The banking sector is undergoing a period of significant transformation. Increased competition, the rise of digital banking, and evolving customer expectations are forcing banks to prioritize customer retention strategies. Retaining existing customers is not only more cost-effective than acquiring new ones, but it also fosters brand loyalty and drives long-term profitability. Understanding the factors influencing customer retention is crucial for banks to develop effective strategies and gain a competitive edge. Management theorists agree upon two crucial points that set customer retention management apart as a vital component of efficient organizational management. First, it is impossible to undervalue the cost of growing one's clientele in these uncertain times. Second, businesses that devote resources to cultivating long-term client connections eventually see increased profits [1]. According to Dawkins and Reichheld [2], in a variety of business settings, even a small 5 % improvement in customer retention might result in a significant increase in net present value, ranging from 25 to 95 %. According to research cited by Boadu and Achiaa [3], keeping customers increases efficiency and lowers costs associated with customer loyalty. Ricadonna, and Saifullah [4], draw attention to the growing interest of academics and business executives in client retention, which has developed from simple retention to a cutting-edge tactic for building loyalty and increasing profits. Despite the obvious advantages of such efforts, there is little indication that financial institutions are using retention strategies [5]. By investigating consumer views on retention techniques, this research seeks to close this knowledge gap by improving comprehension of client retention strategies used in the banking industry.

Direct staff-customer contacts have been the focus of most studies on the relationship between financial performance, customer happiness, and service quality [6]. According to Grima, and Corcoran [7], technological developments have fundamentally changed service marketing dynamics and greatly increased the range of service delivery possibilities. According to Akter, and Gunasekaran [8], service companies may get a competitive advantage by using technology, which not only creates barriers to entry but also improves operational effectiveness and brings in money via creative services.

Furthermore, cultural preferences have a significant role in consumers' acceptance of digital banking services, which has led academics to investigate the social aspect [9]. Khan [10], highlights how different cultural backgrounds have different viewpoints on online banking, with differences between Pakistan and China serving as examples [11]. However, to examine the moderating influence of cultural variations, this research explores the often-ignored relationships between financial technology, consumer trust, and service quality in connection to customer satisfaction, especially in the starkly different settings of China and Pakistan. This study aims to improve the conceptual model's application to the banking sector since previous studies have overlooked some aspects of the banking sectors in these nations. Wewege, and Lee [12], emphasize the need to look into digital banking services beyond national borders to promote regional banking growth.

This study also aims to explore the significance of customer retention tactics in the banking business, to contribute to the Unified Theory of Acceptance and Use of Technology. Despite previous studies demonstrating the crucial role of customer retention in financial performance, there is a scarcity of data about retention methods inside financial institutions. The primary objective of this study is to enhance understanding in this field via the assessment of customer perspectives.

A significant portion of the current body of literature tends to overlook the significance of technology in service delivery, instead prioritizing research that focuses on individual interactions between staff members and clients. This study aims to fill this void by investigating the impact of technology on service quality, customer satisfaction, and trust, specifically in the culturally diverse contexts of Pakistan and China. The primary objective of this study is to provide insight into the influence of cultural variances on the adoption of digital banking services. This research aligns with the need for cross-national comparisons in the field. Ultimately, the objective is to foster the expansion of regional banking and enhance the viability of the conceptual framework within the banking sector.

## 2 Theoretical foundation

### 2.1 Unified theory of acceptance and use of technology (UTAUT2)

The primary inquiry that technology adoption models aim to address pertains to integrating technology into organizational frameworks and individual daily practices. As authorities and financial institutions strive for the full integration of financial technology, this issue has become more significant in promoting digital banking [13]. Venkatesh, and Thong [14] established the Unified Theory of Acceptance and Use of Technology (UTAUT). This model represents an advancement compared to previous ones, elucidating the integration of technology into individuals' everyday routines and the functioning of businesses. To enhance the comprehensiveness of the framework, UTAUT integrated, substituted, or modified several elements from prior prevailing models. UTAUT2, an enhanced iteration of this idea, incorporates additional aspects such as habit, perceived worth, and intrinsic motivation [15]. Scholars have commended the comprehensive nature of UTAUT2, asserting that it encompasses all pertinent attributes that forecast technology adoption [16].

The current study asserts that the UTAUT2 framework is a suitable theoretical framework for examining the proliferation of digital banking in China and Pakistan. The first UTAUT model faced criticism from researchers in the area of information systems due to its failure to consider individuals' distinct technological use patterns [17]. However, this limitation was rectified in the updated and enlarged UTAUT2 model. The improvements expanded the model's breadth, including individual perspectives alongside organizational contexts [18]. Numerous studies have used the Unified Theory of Acceptance and Use of Technology framework to elucidate the factors that drive individuals to engage in action, resulting in their tangible utilization of technology [19]. A comparative analysis of two countries characterized by advanced economies and distinct cultural traditions shows that the UTAUT2 paradigm effectively predicts the uptake of digital banking in diverse cultural and technical contexts [20].

**Fig. 1:**
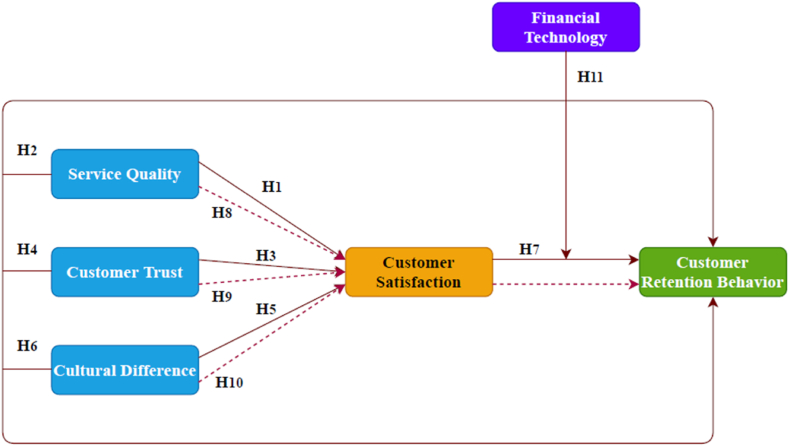
Conceptual model.

To thoroughly examine Exploring Customer Retention Dynamics, it is imperative to consider certain crucial factors. This study aims to understand a comparative investigation of factors affecting customer retention in the banking sector using a Mediation-Moderation Approach. The conceptual model used in this study is illustrated in Fig. 1.

## 3 Literature and hypotheses development

### 3.1 Service quality

Service quality is becoming important in the banking sector in determining customer satisfaction. Boonlertvanich [21], conducted research in which they found many factors for evaluating service quality. This method included creating direct links between service quality characteristics and notions such as satisfaction or loyalty. Rahi and Abd. Ghani [22], identified four core dimensions of SQ, required in an online environment, which include customer service, website design, assurance, and reliability. Alternatively, the research proposed consolidating service quality characteristics into a unified latent service quality variable before linking it with other variables. Contrarily, Hossain, and Jahan [23], arrived at a contrasting determination, saying that contemporary studies in the realm of financial services have conceptualized service quality as a hierarchical, multidimensional construct. Contrary to the preceding method, this viewpoint considers service quality as a hierarchical and multidimensional entity of a higher degree. The formation of this is based on primary service-quality parameters, which are assessed using many survey questions [21,24]. Therefore, the current considers three items to measure service quality as a first-order contract aiming to investigate its direct effect on customer satisfaction and indirection effect on customer retention behavior. According to Torres [25] customer perceptions of quality are believed to be evident at many levels within a service setting. At first, clients evaluate the quality of their relationship with the service provider based on certain attributes. In the banking business, Teeroovengadum [26], contended that the evaluation process advances to the dimensional level, ultimately resulting in a comprehensive assessment of perceived service quality. Furthermore, he stressed that the commonly accepted notion is that perceived quality plays a crucial role in determining consumer happiness. Khatoon, and Zhengliang [27], provide empirical data that strengthens the association between perceived quality and customer happiness, particularly in the banking industry and within certain demographic segments. In addition to consumer happiness, Hardyansah and Jahroni [28], found that service quality is a forerunner to customer satisfaction.

Hence, it proposed that:

H1 Service quality has a positive effect on customer satisfaction

H2 Service quality has a positive effect on customer retention behavior

### 3.2 Customer trust

Trust in customer service, as defined by Franklin [29], refers to the customer's confidence in the service provider's ability to meet their needs and refrain from any detrimental actions. Akram, Abbas [30], customers develop confidence when they see the staff's expertise and promptness, leading them to prioritize trust above other elements of exceptional service. Ferm and Thaichon [31], explored that trust is a crucial component of every connection, as stated by social exchange theory, which serves as the foundation for studying relationships in the banking industry and other fields. A trust may be categorized into two levels: trust in an individual service representative and trust in the banking industry. The previous level encompasses the sense of reliability and benevolence. This confidence in reliability and trustworthiness is referred to as “trust” [32].

Similarly, Khan, and Ali [33] have shown that establishing trust between a service provider and its clients results in enduring loyalty. GEÇİT and TAŞKIN [34], propose that trust plays a significant role in customer loyalty. On the other hand, Damberg, Schwaiger [35], a comprehensive understanding of how trust influences customer trust and the intricate relationship between satisfaction and loyalty may be acquired via this multidimensional definition of trust. Hence, the objectives of the current study are to quantify the effect of customer trust that customers have in banks by defining it as an internal factor that influences the connections between satisfaction loyalty and service quality loyalty. Based on this paradigm, we can now state the following hypotheses:

H3 Customer trust has a positive effect on customer satisfaction.

H4 Customer trust has a positive effect on customer retention behavior.

### 3.3 Cultural difference

Cultural differences can positively impact customer satisfaction in the banking sector, as seen in the context of Pakistan and China [36]. In Pakistan, cultural values such as strong interpersonal relationships and a high level of personal service enhance customer satisfaction by fostering trust and loyalty. Conversely, in China, where efficiency and technological integration are highly valued, banks that leverage advanced digital services and streamlined processes can significantly boost customer satisfaction. By aligning their service approaches with these cultural preferences, banks in both countries can improve their customer experiences and retention. Malik [37], has emphasized the importance of culture. The authors have examined the significant influence of cultural disparities on both consumer loyalty and contentment. Culture is a unified entity with a clear objective, containing a variety of behavioral and distinguishing traits. Furthermore, according to Galariotis and Karagiannis [38], culture may be conceptualized as an intricate framework that includes cognitive processes, affective states, and actions that are deeply rooted in people's convictions and principles. Goswami, and Agrawal [39] define culture as the variation in individuals' behavior across different societies, influenced by a common conceptual framework. Academics contend that the concept of culture, defined as a common framework, encompasses several levels ranging from the national to the individual, specifically including the organizational level within this spectrum [40]. Mingaleva, and Shironina [41], argue that corporations should develop an organizational culture that closely corresponds to the dominant national culture in their operating environment. Although there is a similarity, a difference arises between national and organizational culture, highlighting the interaction of shared ideas, values, and practices among particular groupings of individuals [25]. McSweeney [42], argues that culture is only partly shared among persons who live or have lived in the same social setting. McSweeney [42], makes a substantial contribution to the comprehension of culture by suggesting five characteristics of national culture that impact onimpact society and individuals. Unfortunately, the precise particulars of these dimensions are yet to be explained in the present discussion. Thus, the following hypotheses are presented:

H5 Cultural difference has a positive effect on customer satisfaction.

H6 Cultural difference has a positive effect on customer retention behavior.

### 3.4 Customer satisfaction

Marcos and Coelho [43], customer satisfaction is the overall perception and evaluation that customers develop based on their experience of acquiring or using a product or service. This perception often includes appraisals of the service received after consumption. They further argue that a subjective sensation emerges from the disparity between one's anticipations and real-life encounters. Safitri, and Siregar [44], concluded that constant satisfaction is crucial for achieving long-term contentment with a certain product or service. An assessment of overall satisfaction is a more reliable measure that directly affects customer loyalty towards the service provider. This measure assesses the combined experience of interacting with the provider [45]. Customers' evaluations of service quality are impacted by transactional satisfaction, as shown by a study conducted by Moon and Armstrong [46]. Nevertheless, as shown by Refs. [47,48], the general contentment of customers is seen as an outcome of their perception of quality and serves as a more accurate indicator of their loyalty [49,50]. are recent studies that have opted to assess overall satisfaction using a range of items, in contrast to previous research which often relied on a single item. A research conducted by Alzaydi [51], in which the author employed six elements to evaluate the role of customer satisfaction on the connections among service quality, customer trust, financial technology, and customer retention behavior in the banking industry.

There is a growing trend in banking service research to use models showing how perceived service quality indirectly influences loyalty via satisfaction. This approach has gained popularity and has been studied by researchers such as [52,53]. However, the present study used customer satisfaction as a mediator to examine the association between service quality, customer trust, cultural differences, and customer retention behavior in the banking sector of Pakistan and China.

Hence, we put up these hypotheses:

H7 Customer satisfaction has a positive direct effect on customer retention behavior.

H8 Customer satisfaction mediates the relationship between service quality and customer retention behavior.

H9 Customer satisfaction mediates the relationship between customer trust and customer retention behavior.

H10 Customer satisfaction mediates the relationship between cultural differences and customer retention behavior.

### 3.5 Financial technology

Khan, ul Hassan [54], performed a thorough investigation of the impact of electronic banking on customer satisfaction in the Pakistani setting. The study's results demonstrated a noteworthy correlation between the quality of service and customer satisfaction, with dependability having the greatest influence, followed by responsiveness and assurance. In financial theory, as explained by Fama [55], banking functions as both an accounting system for transactions and a portfolio system for storing assets. The enduring significance of this pivotal function, as emphasized by Fama [55], remains unwavering for the future of the banking industry. Fama [55] argues that in a situation without regulations, banks behave following the Modigliani and Miller [56], theorem, which states that financing choices are not important. Practically, conventional banks compete for deposits by giving different interest rates. Broby [57], explored that as a result, the transactional nature of banks relies on the debits and credits they handle, thus turning them into accounting organizations that serve as intermediaries. Considering that this adjustment is influenced by competitive forces, the overall balance is essentially inactive. Hence, the banking business model is vulnerable to change, especially due to advancements in financial technology.

The findings of a previous study conducted by Tsindeliani, and Proshunin [58] highlight that implementing financial technology infrastructure has greatly transformed the nature and standard of banking operations. In a study conducted by Addai, and Ameyaw [59], a purposive selection technique was used to choose a sample of 150 bank clients from three banks in Ghana. Their research sought to show a correlation between electronic banking and consumer happiness. The results provide strong empirical evidence for the favorable influence of the accessibility, dependability, and ease of electronic banking on consumer contentment. These varied studies provide useful insights into the complex connection between electronic banking, service quality, and consumer happiness in various geographical and cultural settings. Therefore, we proposed that:

H11 Financial Technology moderates the relationship between customer satisfaction and Customer retention Behavior

### 3.6 Customer retention behavior

Boadu and Achiaa [3] define customer retention as the extent to which a current customer maintains a commercial relationship with a financial institute or firm. In the context of a retail commercial bank, this implies that the customer's account is managed and maintained. Based on prior research conducted by Darzi and Bhat [60], maintaining customer retention is a crucial objective for service businesses in relationship marketing. Their growth and survival in a competitive world depend crucially on it. Hawkins and Hoon's [61], comprehensive methodology for estimating client retention incorporates several elements such as overall business satisfaction, positive word of mouth, repeat purchases, and loyalty. Furman, and Diamant [62], argue that a low defection rate is linked to a high retention rate, which is often seen as the opposite of customer defection. Taylor, and Kitchen [63], state that a modest gain of 5 % in customer retention may lead to a substantial 75 % increase in profitability. The author emphasizes the importance of soliciting feedback from clients as a means to enhance service quality, enhance customer loyalty, and augment profits. Fam, Liat Cheng [36], explored that customer retention has been particularly noticeable in the banking industry due to its widespread impact on several parts of life. Over the last decade, the banking industry has seen significant transformations in its organizational framework, degree of rivalry, approaches, plans, and technological landscape. Akdoğan and Özşuca [64] emphasize that the banking industry has seen the impact of globalization, liberalization, deregulation, and technological advancements, which have influenced economies on a global scale. Nyagadza [65], in today's rapidly changing world, banks see the increasing significance of customer retention and the need to adopt innovative strategies to maintain a competitive edge.

## 4 Methodology of the research

The research used a quantitative data collection procedure as a methodological choice [66]. According to Gelo, and Braakmann [67], the primary objective of quantitative research is to enhance our comprehension of the social environment. Researchers use quantitative approaches to systematically examine and comprehend the impact of events or conditions on individuals within a certain context. Statistical analyses and numerical representations provide a concise and unambiguous explanation of findings in quantitative research, yielding objective data. This technique enables researchers to get significant insights and address knowledge gaps in social dynamics.

### 4.1 Data collection method and selection of samples

The data collection for this study used a survey methodology that involved a structured questionnaire. This type of selection is also beneficial in reducing the work burden and costs that would have been involved in studying the entire target population [68]. The survey instrument included a questionnaire that profiled the participants and included pivotal questions that encapsulated all the significant aspects. According to Rahman [69], the quantitative survey method helps reveal aspects related to the emotions, behaviors, and perceptions of respondents, exploring dimensions beyond numerical representation. The survey was conducted in three prominent Chinese cities, including Beijing, Shanghai, and Guangzhou, as well as two capitals in Pakistan, Rawalpindi and Gilgit. The target demographic included graduate and postgraduate students from five Pakistani universities and five Chinese mainland institutions. Considering the students' proficiency in the complexities of digital banking, prior research has shown their responsiveness and authenticity. These two countries were selected based on their mutual cultural history, robust economic ties facilitated by the China-Pakistan Economic Corridor, and their shared commitment to collaborating on other issues [70]. The survey questionnaire used convenience sampling that is close at hand and easily accessible to the researcher to ensure impartiality and equity in participant selection, enhancing the study's robustness. Convenience sampling offers a cost-effective method for conducting research or collecting responses. However, this approach is susceptible to selection bias as the sample may not accurately represent the target population [71].

Before the widespread distribution of the questionnaire, a focus group was formed to assess the questions, their interpretations, context, and content [72]. The instrument underwent meticulous adjustments to enhance its clarity and coherence, considering the perceptive remarks provided by a focus group of researchers and relevant experts. By doing this, we intended to minimize any potential for ambiguity in the survey. The target respondents were university students and individuals with a comprehensive understanding of digital banking services and holding active bank accounts. Information was collected using a combination of tangible and digital approaches. In addition to paper surveys, an online survey link was sent via several social media platforms and email addresses, including WeChat, QQ, WhatsApp, and Facebook [73]. 593 questionnaires were distributed in both China and Pakistan. We offered modest incentives to incentivize engagement and explicitly communicated to participants that their responses were voluntary. The focus was placed on ensuring participant anonymity and data confidentiality, with the assurance that the information would only be used to analyze digital banking in Pakistan and China.

According to Dawes's [74], recommendations, the current study used a five-point Likert scale to promote active engagement among individuals. This five-point Likert scale will span from “strongly disagree” (1) to “strongly agree” (5). The surveys will be distributed by proficient surveyors who will be strictly supervised by the research team [75]. Finally, the data was analyzed using partial least squares structural equation modeling (PLS-SEM4) [76].

### 4.2 Measurement items

**Table 1:** Measurement items.

Name of Variable	Items	References
Service Quality	The financial institution has a clear and transparent service routine, which helps develop its reputation for being reliable.	[21]
	The bank has established a rigorous method and system to effectively handle client data safely.	
	The Bank guarantees the precision of its comprehensive service method.	
	Service officers efficiently provide easy services, reducing any complex procedures for clients.	
Customer Trust	The availability of my bank manager in difficult circumstances is constantly trustworthy.	[78]
	The bank manager regularly makes beneficial suggestions to facilitate the development of my firm.	
	The advice given by my bank manager enhances my trust in my decision-making process.	
	I have confidence in my bank manager's competence to aggressively respond to and adjust to the changing financial needs of my firm.	
Cultural difference	The cultural characteristics in Pakistan have a crucial impact on enhancing customer satisfaction and encouraging the long-term use of financial services.	[79]
	The Chinese cultural backdrop has a vital role in improving customer satisfaction and promoting the long-term use of financial services.	
	The promotion of ethical and cultural values in financial operations has a substantial impact on customer decision-making in the banking industry.	[80]
	The bank's strong engagement in charitable efforts and community initiatives positively impacts customer retention and devotion.	
Customer Satisfaction	I made a wise choice when selecting this service provider.	[81] [21]
	The bank continuously fulfills my expectations by providing services of exceptional quality	
	Overall, my experience with this banking institution has been acceptable.	
	Overall, I feel satisfied with this bank	
Financial technology	The effectiveness of Internet transactions enhances my trust in the bank.	[79]
	I expect that the performance of Internet banking will be comparable to that of other technologies, such as telephone or TV banking.	
	I had a strong belief in the dependability of online banking, and it lived up to my expectations.	
	Utilizing financial technology enables me to conveniently reach my bank anytime, boosting flexibility during business hours.	
Customer retention behavior	Customer feedback is systematically gathered on a regular schedule every week.	[60]
	Personalized communications contribute to my perception of success, cultivating strong connections with the bank.	
	The bank fosters a culture that places client satisfaction as the highest priority.	
	The bank organizes regular client meetings to promote participation and communication.	

## 5 Analysis

The current study used structural equation modeling (SEM) with smart-PLS to extract results from both structural and measurement models. Smart-PLS is a dependable and effective analytical technique used for extracting data related to variables. It is considered superior to other third-generation techniques [77]. Employing Structural Equation modeling modeling (SEM) with smart-PLS is a practical and advantageous approach, particularly in situations that need predictive modeling. Smart-PLS simplifies the usage of many analytical methods such as principal component analysis, reliability and validity evaluations, multivariate regression, and complicated model assessments [76] (see Table 1).

### 5.1 Respondents’ information

The participants' demographic characteristics are shown in Table 2. Factors to consider include age, education, gender, and years of professional experience, among other factors. Upon examining the statistical data from Pakistan, it is evident that 71 % of males and 28.8 % of women possess the ability to get financial services. According to the study findings, 57.6 % of the male participants and 42.3 % of the female participants in the Chinese sample use the Internet and can get financial services. The majority of participants in both the Chinese and Pakistani groups had obtained bachelor's degrees or above in terms of their educational level. In Pakistan, 53.7 % of the participants are between the age ranges of 26–35, whereas in China, this percentage is 60.8 %. Furthermore, a significant proportion of Chinese and Pakistani participants, ranging from 42 % to 54.4 %, reported having experience as bank account holders in the banking sector for one to five years. The findings indicate that the participants' demographics were diverse and represented.

**Table 2:** Demography of the respondents.

Pakistani Samples: 281			Chinese Samples: 312
Sex	Male	200	180
	Female	81	132
Age	18–25	50	50
	26–35	151	190
	36–50	80	72
Profession	Student	100	132
	Businessmen	181	180
Education	BA	100	110
	Masters	160	160
	Ph.D.	21	42
Experience of Bank Account Holders	1–5	130	170
	6–10	126	131
	11–15	25	11

### 5.2 Assessment of measurement model

The SEM enables the analysis of two models: the measurement (outer) model and the structural (inner) model [82]. The measuring approach specifically entails evaluating the reliability and validity of latent constructs via the examination of factor loadings, Cronbach's alpha statistics, composite reliability (CR), and average variance extracted (AVE) [83]. The results of the measuring model demonstrate strong content, convergent, and discriminant validities, satisfying the predetermined requirements. As shown in Table 3, all factor loadings exceed the suggested threshold of 0.70 [84]. Furthermore, the measures' reliability and validity, as assessed by CR and AVE values, meet the acceptable thresholds of 0.70 for CR and 0.50 for AVE [85]. Table 3 demonstrates the sufficiency of CR (composite reliability) and AVE (average variance extracted) by displaying values higher than 0.70. The scale dependability indicates a strong and appropriate model, with Average Variance Extracted (AVE) values over 0.50, which suggests significant convergent validity. Henseler, and Ringle [86], propose that an average variance extracted (AVE) value greater than 0.50 indicates a reasonable level of variation for the construct.

**Table 3:** Measurement of factor loadings and reliability (Pakistan).

Constructs	Items	Outer Loadings	Cronbach's Alpha (CA)	Composite Reliability (CR)	Average Variance Extracted (AVE)
Service Quality (SQ)	SQ1 SQ 2 SQ 3 SQ 4	0.793 0.812 0.819 0.770	0.811	0.874	0.696
Customer Trust (CT)	CT1 CT2 CT3 CT4	0.788 0.786 0.812 0.752	0.792	0.741	0.553
Cultural Difference (CD)	CD1 CD2 CD3 CD4	0.830 0.848 0.838 0.821	0.857	0.800	0.616
Customer Satisfaction (CS)	CS1 CS 2 CS 3 CS 4	0.733 0.792 0.759 0.852	0.792	0.793	0.616
Customer Retention Behavior (CRB)	CRB 1 CRB 2 CRB 3 CRB 4	0.737 0.750 0.708 0.779	0.733	0.821	0.595
Financial Technology (FT)	FT1 FT2 FT3 FT4	0.742 0.728 0.746 0.862	0.777	0.811	0.638

In contrast, Table 4 provides a detailed analysis of the Chinese sample, specifically focusing on outer loadings, Cronbach's alpha (CA), composite reliability (CR), average variance extracted (AVE), and the relationships between components in terms of their strength and direction. Both the CA and CR values are above the predefined threshold of 0.70, indicating a high level of composite dependability. Furthermore, the AVE values regularly reach or surpass 0.50, confirming the positive convergent validity of the measurements. Therefore, Table 4 illustrates that all AVE values satisfy the specified threshold level.

**Table 4:** Measurement of factor loadings and reliability (China).

Constructs	Items	Outer Loadings	Cronbach's Alpha	Composite Reliability (CR)	Average Variance Extracted (AVE)
Service Quality (SQ)	SQ1 SQ 2 SQ 3 SQ 4	0.803 0.816 0.816 0.766	0.834	0.838	0.666
Customer Trust (CT)	CT1 CT2 CT3 CT4	0.773 0.793 0.801 0.772	0.819	0.818	0.651
Cultural Difference (CD)	CD1 CD2 CD3 CD4	0.812 0.815 0.811 0.827	0.792	0.795	0.617
Customer Satisfaction (CS)	CS1 CS 2 CS 3 CS 4	0.756 0.783 0.768 0.833	0.793	0.794	0.616
Customer Retention Behavior (CRB)	CRB 1 CRB 2 CRB 3 CRB 4	0.837 0.851 0.827 0.704	0.925	1.141	0.805
Financial Technology (FT)	FT1 FT2 FT3 FT4	0.947 0.888 0.900 0.851	0.813	0.813	0.641

#### 5.2.1 Discriminant validity (Heterotrait-Monotrait ratio of correlations) for Pakistani samples

The research used the Heterotrait-Monotrait Ratio of Correlations to assess discriminant validity, as shown in Table 5. According to Ref. [87], if the HTMT value is much lower than the crucial threshold of 0.9, it suggests that there is discriminant validity. The study determined that the HTMT ratios for each pair of constructs were statistically significant. The results shown in Table 5 indicate that the maximum HTMT value found is 0.882, which is lower than the commonly accepted threshold of 0.90. This confirms that the constructs have discriminant validity.

**Table 5:** Discriminant validity (HTMT) (for Pakistan).

Constructs	CD	CRB	CS	CT	FT
CD					
CRB	0.365				
CS	0.753	0.643			
CT	0.754	0.608	0.848		
FT	0.165	0.292	0.229	0.155	
SQ	0.736	0.64	0.809	0.886	0.161
					

#### 5.2.2 Discriminant validity (Heterotrait-Monotrait ratio of correlations) for Chinese samples

Table 6 provides data supporting the development of discriminant validity, which is an important criterion to guarantee that the studied constructs differ. The table demonstrates that each construct retains its character and does not have abnormally high correlations with other constructs in the research. All the values of the Heterotrait-Monotrait Ratio Of Correlations (HTMT) shown in Table 6 satisfy the specified threshold values, as advised by Franke and Sarstedt [87]. This indicates that the constructs examined in the research are sufficiently different from one another, hence strengthening the validity of the analysis in terms of distinguishing between them.

**Table 6:** Discriminant validity (HTMT) (for China).

Constructs	CD	CRB	CS	CT	FT
CD					
CRB	0.656				
CS	0.757	0.861			
CT	0.703	0.861	0.863		
FT	0.073	0.088	0.096	0.128	
SQ	0.590	0.847	0.827	0.876	0.057

#### 5.2.3 Discriminant validity (Fornell-Larker criterion) for Pakistani and Chinese samples

The Fornell-Larcker criteria is a commonly used approach to evaluate the discriminant validity of measurement models [88]. According to the guidelines provided by Henseler, and Ringle [86], the square root of the average variance extracted (AVE) for a construct should be greater than the correlation between that construct and any other construct in the model. Put simply, the fundamental basis of the AVE for a certain concept should exceed its association with all other concepts. Therefore, the results shown in Table 7, Table 8 demonstrate that the Fornell-Larcker criteria have been satisfied for both the datasets from Pakistan and China. These results confirm that all measures used in this research have good levels of discriminant validity (see Table 9).

**Table 7:** Discriminant validity (Fornell-Larcker criterion) (for Pakistan).

Constructs	CD	CRB	CS	CT	FT	SQ
CD	0.834					
CRB	0.315	0.744				
CS	0.643	0.502	0.785			
CT	0.636	0.478	0.674	0.785		
FT	0.047	0.233	0.133	0.066	0.771	
SQ	0.621	0.509	0.649	0.709	0.087	0.799

**Table 8:** Discriminant validity (Fornell-Larcker criterion) (for China).

Constructs	CD	CRB	CS	CT	FT	SQ
CD	0.816					
CRB	0.548	0.807				
CS	0.619	0.697	0.785			
CT	0.577	0.698	0.686	0.785		
FT	−0.059	−0.093	−0.082	−0.101	0.897	
SQ	0.489	0.693	0.665	0.704	−0.054	0.8

**Table 9:** Discriminant validity HTMT (combined: N = 593).

Constructs	CD	CRB	CS	CT	FT
CD					
CRB	0.519				
CS	0.75	0.767			
CT	0.726	0.748	0.862		
FT	0.066	0.089	0.082	0.071	
SQ	0.661	0.758	0.828	0.881	0.054

##### 5.2.3.1 The Heterotrait-Monotrait ratio of correlations used to assess discriminant validity (Combine data for Pakistan and China)

The Heterotrait-Monotrait ratio of correlations (HTMT) is a statistical method used to assess discriminant validity in business management research. It is generally accepted that discriminant validity is acceptable at a level less than 0.90 Henseler, Ringle [86], The HTMT measurements in Table 9 are all below the threshold of 0.90. This implies that the establishment of discriminant validity has been successfully achieved between the two constructs that were assessed reflectively (see Table 10).

##### 5.2.3.2 Fornell-Larcker criterion of correlations used to assess discriminant validity (Combine data for Pakistan and China)

Following the guidelines outlined, it is recommended that the square root of the average variance extracted (AVE) for a construct surpasses its correlation with any other construct in the model. In essence, the AVE should outweigh its associations with all other concepts. Thus, the outcomes depicted in Table 10 affirm fulfilling the Fornell-Larcker criteria (see Table 11).

**Table 10:** Discriminant validity (Fornell-Larcker criterion).

Constructs	CD	CRB	CS	CT	FT	SQ
CD	0.826					
CRB	0.437	0.778				
CS	0.624	0.609	0.785			
CT	0.603	0.595	0.684	0.785		
FT	−0.029	0.085	−0.012	−0.039	0.800	
SQ	0.554	0.608	0.664	0.706	0.003	0.799

**Table 11:** Assessment of R-square and LV prediction summary (Q^2^).

Pakistani Samples			Chinese Samples	
Variables	R-square	Q^2^predict	R-square	Q^2^predict
CRB	0.363	0.300	0.623	0.574
CS	0.559	0.541	0.593	0.577

### 5.3 Assessment of structural model

**Fig. 2:**
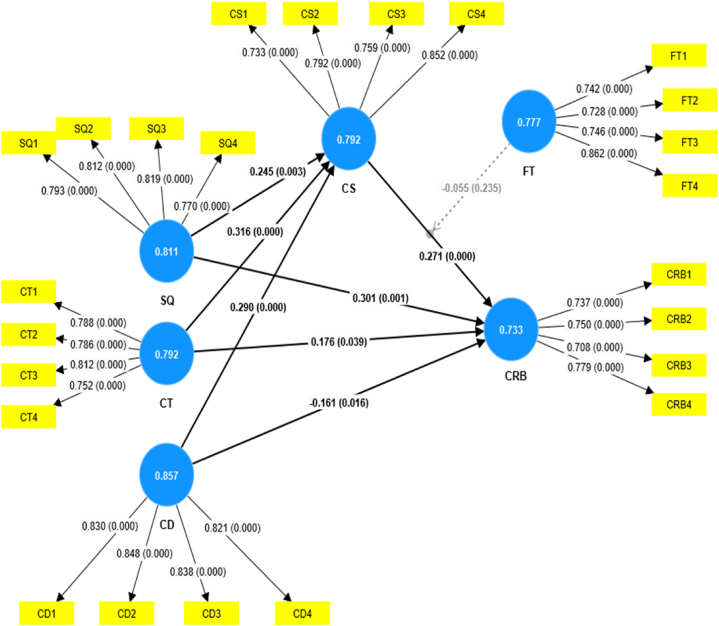
SEM model for Pakistani samples.

**Fig. 3:**
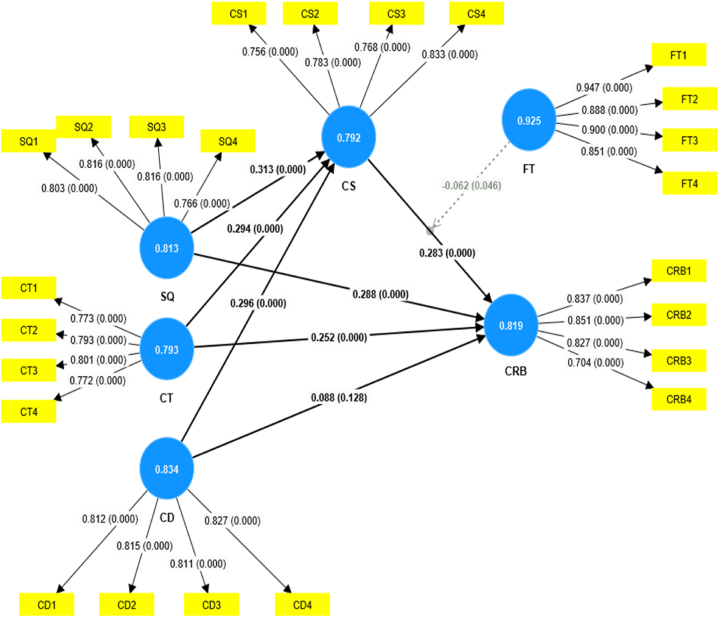
SEM model for Chinese samples.

The structural equation modeling (SEM) approach involves evaluating the structural or inner model, which reveals the links between different constructs, after obtaining positive results from the measurement model. Fig. 2, Fig. 3 demonstrate the outer models for Pakistan and China, respectively, highlighting the suitability and appropriateness of the measures. After achieving good findings from the measuring model, the author performed the structural model evaluation to examine the links between the constructs.

Leroi-Werelds, and Streukens [89], consider the use of the R2 coefficient, also referred to as the coefficient of determination, in PLS-SEM to evaluate measurement variance. The R2 statistic quantifies the amount of variation in the dependent variable that can be accounted for by the independent variable. Falk and Miller [90], assert that R2 values should be equal to or greater than 0.10 to be deemed adequate for explaining the variation in a certain endogenous concept. On the other hand, Cohen [91], proposes evaluating R2 values for endogenous latent variables as follows: 0.26 (significant), 0.13 (moderate), 0.02 (poor). Table 9 shows that the R square values for Pakistan are statistically significant, with a value of 0.36 for CRB and 0.559 for CS. In contrast, the R square values for China for CRB and CS are 0.623 and 0.593, respectively.

The Q-square metric evaluates the predictive significance of a model by determining its capacity to make accurate predictions. Consistent with Chin [92], Q-square values greater than zero indicate accurately reconstructed values and confirm the model's predictive significance. Notably, a Q2 value greater than zero implies the existence of predictive significance. Upon analyzing Table 11, it is clear that the Q-square values for the Pakistani sample, notably for CRB (0.300) and CS (0.541), as well as for the Chinese sample, with Q-square values for CRB (0.574) and CS (0.577), are all considerably more than zero. This finding indicates that the model has excellent prediction accuracy (see Table 12).

#### 5.3.1 R-squared in regression analysis and Mv prediction summary (combined data for Pakistan and China)

The coefficient of determination (R2) is used to quantify the degree to which the independent variable accounts for the variability seen in the dependent variable. According to Falk and Miller [90], R2 values fall within the range of 0–1, where values closer to 1 indicate greater prediction accuracy. The present research utilizes the R2 threshold values established by Cohen [91], whereby an R2 value of 0.26 is considered significant, 0.13 is considered moderate, and 0.02 is considered weak. The R-square values for Pakistan and China are statistically significant, with CRB at 0.482 and CS at 0.578, as shown in Table 12.

**Table 12:** R-squared in regression analysis (combined data for Pakistan and China).

Variables	R-square
CRB	0.482
CS	0.578

Conversely, the Q-square measure assesses the predictive relevance of a model by evaluating its capacity to provide precise predictions (see Table 13). By Chin's, findings, Q-square values that exceed zero indicate accurate reconstructions and validate the predictive validity of the model. Significantly, a Q2 score greater than zero indicates the presence of predictive relevance. Table 13 reveals significant Q-square values for both Pakistan and China, namely for CRB (0.363) and CS (0.559), which are significantly higher than zero. These results highlight the remarkable prediction accuracy of the model.

**Table 13:** Mv prediction summary (combined data for Pakistan and China).

Variables	Q^2^predict
CRB	0.431
CS	0.571

##### 5.3.1.1 Testing hypotheses

According to Hair Jr, Hult [93], Structural Equation Modelling is a collection of statistical methods specifically developed to analyze the relationships between observable and latent variables. Semantic analysis, or SEM, surpasses the limitations of regression analysis by assessing linear causal connections between variables while including measurement errors. This research evaluated The suggested model using Structural Equation Modelling in PLS 4.0. Fig. 2, Fig. 3 provide a visual representation of the model. The route coefficient analysis technique was used to test the alignment of the model with the dataset and to evaluate the interrelationships between variables simultaneously. To get reliable outcomes, a bootstrapping technique was used, as suggested for achieving the best possible results when working with small to moderate sample sizes.

The hypotheses testing has been performed by examining the beta values, standard deviation, T-statistics, and P- values. According to Table 14, Pakistani samples illustrate that quality service significantly affects customer satisfaction at p values = 0.003 and 0.000. This shows that H1 is supported for Pakistani and Chinese samples. Results also specify that quality service significantly affects customer retention behavior P-values 0.001 and 0.000, which provides strong evidence in favor of H2 for Pakistani and Chinese samples. According to Table 12, customer trust significantly affects customer satisfaction p-0.000 and 0.000, hence providing support for H3 for both Pakistani and Chinese samples. Similarly, customer trust has a positive effect on customer retention behavior with p-values of 0.039 and 0.000 showing that H4 is supported in the context of both countries. Results depict that cultural differences have had a positive effect on customer satisfaction P-values 0.000 and 0.000, provide strong evidence in favor of H5 for Pakistani and Chinese samples. Analysis of Pakistani samples reveals that cultural difference significantly affects customer retention behavior P-value 0.016, thus H6 is supported. On the other hand, results from Chinese samples show that cultural difference has an insignificant impact on customer retention behavior P-value 0.128; hence, H6 is not supported. Results of Pakistani and Chinese samples show that customer satisfaction positively affects customer retention behavior with P-values 0.000 and 0.000. Thus, H7 is supported (see Table 15).

**Table 14:** Path-coefficient and hypotheses analysis.

For Pakistani Samples						For Chinese Samples				
Constructs	Beta Values	Standard deviation	T statistics	P values	Remarks	Original sample	Standard deviation	T statistics	P values	Remarks
SQ - > CS	0.245	0.083	2.941	0.003	Supported	0.313	0.070	4.455	0.000	Supported
SQ - > CRB	0.301	0.087	3.461	0.001	Supported	0.288	0.062	4.661	0.000	Supported
CT - > CS	0.316	0.078	4.044	0.000	Supported	0.294	0.067	4.415	0.000	Supported
CT - > CRB	0.176	0.086	2.062	0.039	Supported	0.252	0.064	3.918	0.000	Supported
CD - > CS	0.290	0.06	4.801	0.000	Supported	0.296	0.053	5.569	0.000	Supported
CD - > CRB	−0.161	0.067	2.408	0.016	Supported	0.088	0.058	1.520	0.128	Not Supported
CS - > CRB	0.271	0.077	3.499	0.000	Supported	0.283	0.062	4.56	0.000	Supported

##### 5.3.1.2 Mediation and moderation tests

Table 15 shows the mediation and moderation test for Pakistani and Chinese samples. Upon analysis of Table 15, it becomes evident that customer satisfaction significantly mediates the relationship between service quality and customer retention behavior. This is substantiated by the presence of noteworthy P-values of 0.015 for Pakistani samples and P-values of 0.001 for Chinese samples. Consequently, this outcome offers extensive validation for hypothesis 8. Results reveal a positive mediating effect of customer satisfaction on the relationship between customer trust and customer retention behavior. Thus, hypothesis 9 receives strong support, as shown by the remarkable P-values of 0.013 for Pakistan and 0.003 for China. Regarding hypothesis 10, which suggests a positive mediation effect of customer satisfaction on the relationship between cultural difference and customer retention behavior, the empirical evidence supports this claim with P-values of 0.009 for Pakistani samples and 0.000 for Chinese samples. Hence, H10 is supported (see Table 16).

**Table 15:** Mediation and moderation analysis.

Mediation Test for Pakistan						Mediation Test for China				
Constructs	Beta Values	Standard deviation	T statistics	P values	Remarks	Beta Values	Standard deviation	T statistics	P values	Remarks
SQ - > CS - > CRB	0.066	0.027	2.434	0.015	Supported	0.088	0.027	3.223	0.001	Supported
CT - > CS - > CRB	0.086	0.035	2.474	0.013	Supported	0.283	0.028	2.99	0.003	Supported
CD - > CS - > CRB	0.079	0.030	2.608	0.009	Supported	0.084	0.024	3.502	0.000	Supported
**Moderation Test for Pakistan**						**Moderation Test for China**				
**Constructs**	**Beta Values**	**Standard deviation**	**T statistics**	**P values**	**Remarks**	**Beta Values**	**Standard deviation**	**T statistics**	**P values**	**Remarks**
FT x CS - > CRB	−0.055	0.046	1.188	0.235	Not Supported	−0.062	0.031	1.996	0.046	Supported

**Fig. 4:**
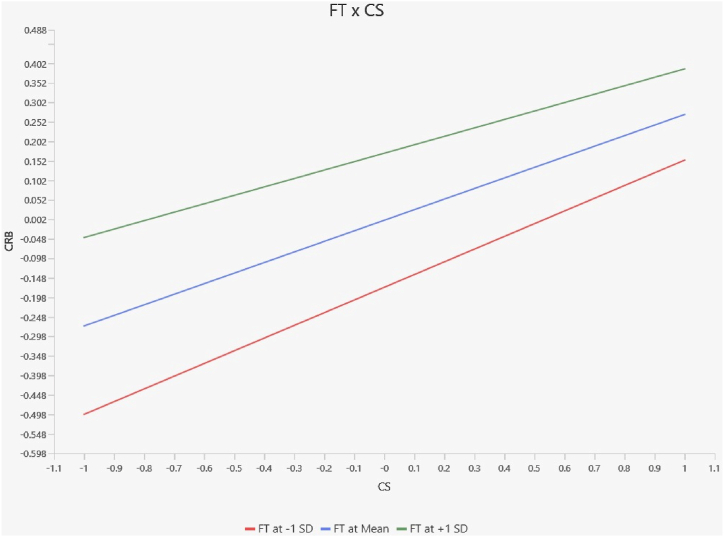
Moderation slope analysis for Pakistan.

**Fig. 5:**
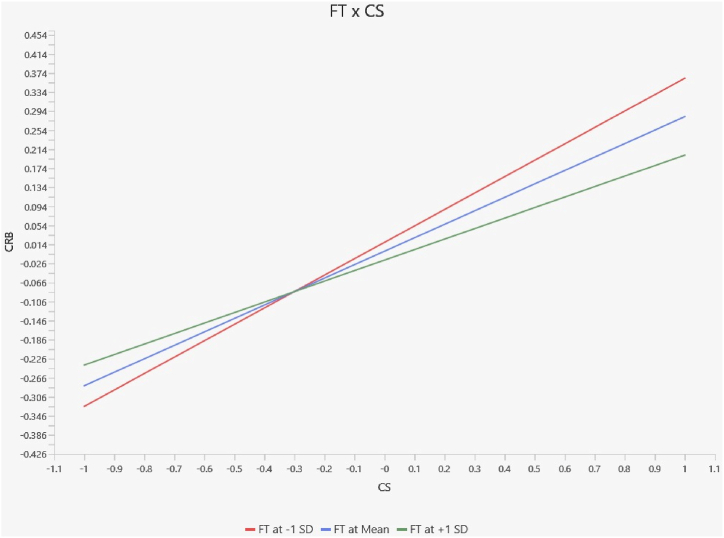
Moderation slope analysis for China.

Table 14 also illustrates that H11 financial technology negatively moderates the relationship between customer satisfaction and customer retention behavior, as shown by insignificant P-values of 0.235 for Pakistani samples (Fig. 4). On the other hand, H11 receives strong support, as demonstrated by the significant P-values of 0.046 for Chinese samples. Hence, the analysis supports hypothesis 11 in the context of China (Fig. 5).

Partial Least Squares Structural Equation Modeling (PLS-SEM) was used to examine hypotheses about combined data for Pakistan and China. The study of beta coefficients, standard deviations, T-statistics, and P-values aided this testing. The findings shown in Table 16 provide evidence of a substantial relationship between service quality and customer satisfaction. The beta coefficients for this relationship are 0.292, the T-statistics are 5.493, and the P-values are 0.000. These results support hypothesis H1. Furthermore, the study revealed a substantial relationship between service quality and customer retention behavior. This is supported by beta coefficients of 0.275, T-statistics of 5.439, and P-values of 0.000, providing strong evidence in favor of hypothesis H2 (see Table 17).

**Table 16:** Path-coefficient and hypotheses testing for Pakistan and China (pooled: N = 593).

Constructs	Beta Values	Standard deviation	T statistics	P values
SQ - > CS	0.292	0.053	5.493	0.000
SQ - > CRB	0.275	0.05	5.439	0.000
CT - > CS	0.312	0.052	5.981	0.000
CT - > CRB	0.221	0.051	4.34	0.000
CD - > CS	0.273	0.039	7.023	0.000
CD - > CRB	−0.027	0.045	0.597	0.551
CS - > CRB	0.299	0.05	6.047	0.000

Additional examination reveals a significant impact of customer trust on customer satisfaction, as shown by beta coefficients of 0.312, T-statistics of 5.981, and P-values of 0.000. These findings provide support for hypothesis H3. The effect of customer trust on customer retention behavior is positive, as evidenced by beta coefficients of 0.221, T-statistics of 4.340, and P-values of 0.000. These findings confirm hypothesis H4. The findings indicate a positive relationship between cultural differences and customer satisfaction. This is supported by the beta coefficients of 0.273, T-statistics of 7.023, and P-values of 0.000, providing substantial evidence favoring hypothesis H5. Nevertheless, the impact of cultural differences on customer retention behavior was shown to be statistically negligible, as indicated by beta coefficients of −0.292, T-statistics of 0.597, and P-values of 0.551.

Consequently, hypothesis H6 was insignificant. In conclusion, the findings suggest a substantial relationship between customer satisfaction and customer retention behavior, as shown by beta coefficients of 0.299, T-statistics of 6.047, and P-values of 0.000. These results provide support for hypothesis H7.

Analysis of Table 17, which presents the mediation and moderation tests on the combined data from Pakistani and Chinese samples, reveals significant insights. The results of Table 16 confirm that customer satisfaction mediates the association between service quality and customer retention behavior. This is evidenced by significant beta values (β = 0.088), T-statistics (4.315), and P-values (0.000), thus providing substantial support for hypothesis 8. Furthermore, the data indicate a positive mediating effect of customer satisfaction on the relationship between customer trust and customer retention behavior, strongly endorsing hypothesis 9 through notable beta values (β = 0.093), T-statistics (4.059), and P-values (0.000). Additionally, support for hypothesis 10 is established, showing that customer satisfaction positively mediates the link between cultural differences and customer retention behavior, as demonstrated by beta values (β = 0.082), T-statistics (4.392), and P-values (0.000).

**Table 17:** Mediation and moderation analysis (pooled: N = 593).

Mediation Test					
Constructs	Beta Values	Standard deviation	T statistics	P values	Remarks
SQ - > CS - > CRB	0.088	0.020	4.315	0.000	Supported
CT - > CS - > CRB	0.093	0.023	4.059	0.000	Supported
CD - > CS - > CRB	0.082	0.019	4.392	0.000	Supported
**Moderation Test for Pakistan**					
**Constructs**	**Beta Values**	**Standard deviation**	**T statistics**	**P values**	**Remarks**
FT x CS - > CRB	−0.097	0.044	2.213	0.027	Supported

More importantly, Table 17 indicates that financial technology significantly moderates the relationship between customer satisfaction and customer retention behavior, as indicated by T-statistics (2.213) and P-values (0.027), thus firmly supporting hypothesis 11. These findings collectively underscore the nuanced roles that customer satisfaction and financial technology play in shaping customer retention behavior.

## 6 Findings and discussion

The current study investigated the effects of service quality, customer trust, and cultural differences on customer satisfaction. It also investigates the mediating effect of customer trust on the relationship between service quality, customer trust, cultural differences, and customer retention behavior. This study also examined the moderating effect of financial technology on the relationship between customer satisfaction and customer retention behavior. For that purpose, we used 281 samples for Pakistan and 312 for China. The study's findings proved that service quality positively affects customer satisfaction in Pakistan and China. These results align with the previous research conducted by Saraswati [94], which provides further evidence that the observed correlation is valid in a wide range of service quality and customer satisfaction. In line with previous research, the results show that service quality is the most critical factor in banking customers' satisfaction. This study found service quality as a stronger predictor of customer satisfaction in Pakistan and China's banking sectors. This indicates that service quality plays a significant role in determining customer satisfaction.

Consequently, to establish strong interpersonal and social connections with their customers, our findings suggest that banks must prioritize their delivery of high-quality service. These results are further substantiated by the conclusions in earlier studies [95]. They concluded that by satisfying customer expectations through quality of services, banks can foster trust among customers and enhance their brand reputation in the highly competitive banking industry. This aligns with the prevailing belief that improving the quality of service banks offer significantly enhances customer satisfaction and cultivates stronger ties between banks and their customers.

This research also highlights the significant effect of quality service on customer retention behavior in Pakistan and China's banking industries. This supports previous studies, such as the study by Alshamsi, and Alshurideh [96], which showed a significant effect of service quality on customer retention in the banking sector. Our research's positive and noticeable impact is consistent with the larger consensus that contented customers are more inclined to maintain a devoted and ongoing affiliation with a bank. Hence, our research adds to the increasing body of data emphasizing the general significance of providing high-quality service to promote customer devotion and retention in the banking industry. The findings underscore the need to prioritize and improve service quality for banks in Pakistan and China, as they face a more competitive environment. This is crucial for building long-term client connections and achieving sustainable commercial success.

Similarly, customer trust has a positive effect on customer satisfaction in the context of Pakistan and China. Our study validates prior research by demonstrating a positive correlation between customer trust and satisfaction. Our findings align with the Rafiq, Jun [97] study, which also observed positive correlations in the banking business. The association between customers' trust in banks and their overall impressions and satisfaction with these services is well established, highlighting the significance of the former in determining the latter. According to Zhang, and Siyal [98], customers exhibit higher satisfaction and loyalty toward a bank when they perceive a sense of trust in their interactions. The findings emphasize the global importance of establishing and maintaining customer trust to enhance overall satisfaction and positive relationships, particularly for banks operating in diverse cultural and economic environments.

The current study's findings confirmed that customer trust positively affects customer retention behavior. Our analysis confirms a positive correlation between customer trust and customer retention behavior in the Pakistan and Chinese banking setting, consistent with previous research in this area. The results align with the research conducted by Alketbi, and Alshurideh [99], who also discovered the positive influence of consumer trust on customer retention in the banking industry. This highlights the cross-cultural uniformity of the correlation between customer trust and retention, indicating that when consumers sense a strong degree of trust in their contacts with banks in China, it increases their probability of staying loyal. The study conducted by Ref. [100] highlights the global importance of trust in promoting enduring connections and client loyalty in the Chinese banking industry. Consequently, our research adds to the expanding body of data demonstrating the strategic relevance of building and retaining customer trust for fostering sustained customer retention in the Chinese banking industry.

The results of our study demonstrate a significant association between cultural differences and customer satisfaction in the banks in Pakistan and China. These findings align with the research conducted by Endara, Ali [101] who also discovered a positive influence of cultural disparities on customer satisfaction within the banking sector. The results emphasize the intricate influence of cultural differences on consumer satisfaction in various settings, namely in our research conducted in Pakistan and China. The study by Lee and Kang [102] emphasizes the significance of recognizing and comprehending cultural subtleties in providing services that align with consumers' cultural inclinations and anticipations. It highlights the importance for businesses, especially in diverse markets such as Pakistan and China, to customize their strategies to acknowledge and value cultural disparities, leading to improved customer satisfaction.

Our study also reveals a significant effect of cultural differences on customer retention in the banking industry in Pakistan, but it is insignificant in China. Endara, and Ali [101], argue that if researchers continue just to link culture with a specific nation, it would impede progress in comprehending the impact of cultural influences. This means that cultural differences confirm prior investigations consistent with the findings of Fam, and Liat Cheng [36], who also found that cultural variations had a beneficial effect on client retention in the banking sector. The results emphasize the need to acknowledge and adapt to cultural subtleties in banking services across different cultures. The current study's findings provide evidence that acknowledging and adjusting to cultural differences have a significant role in fostering more robust and enduring customer connections, eventually impacting their loyalty and retention patterns. Hence, our research contributes empirical data to the current pool of knowledge, highlighting the strategic importance of considering cultural aspects when developing retention strategies for banks operating in culturally varied environments.

The results of our study reveal that customer satisfaction has a positive effect on customer retention behavior in the context of Pakistan and China, which supports existing research and is consistent with the study undertaken by Arshad Khan and Alhumoudi [103], which emphasized the strong influence of customer satisfaction on customer retention. The established connection highlights the essential function of customer pleasure as a catalyst for promoting loyalty and motivating clients to maintain their affiliation with a firm. The research conducted by Almohaimmeed [104] establishes a theoretical basis for our results, highlighting the long-lasting impact of consumer pleasure on future retention behavior. Within our research framework, this signifies that contented customers are more inclined to display actions that contribute to their ongoing involvement with the bank. Therefore, our research adds to the current discussion by providing empirical evidence that confirms the lasting connection between customer happiness and customer retention behavior. This reaffirms the strategic significance of giving priority to customer satisfaction in the banking industry.

### 6.1 Mediating role of customer satisfaction

The results of our study emphasize the significance of customer satisfaction in mediating the link between service quality and customer retention behavior in the context of Pakistan and China, which is in line with the previous study by Arshad Khan and Alhumoudi [103] research, which highlighted the role of customer satisfaction as a mediating factor in shaping future consumer behaviors. Arshad Khan and Alhumoudi's [103], conceptualization offers theoretical support for our observed mediation effect, emphasizing that the effect of service quality on customer retention is transmitted through the perspective of customer satisfaction. However, our findings provide empirical data that supports the theoretical framework. It confirms that service quality's impact on customer retention mostly depends on customer satisfaction. Hence, this study provides evidence and expands upon the current body of research, confirming the intricate relationship between service quality, customer satisfaction, and customer retention behavior within the banking industry.

The results of our present investigation elucidate the mediation effect of customer satisfaction in the connection between customer trust and customer retention behavior in the banking industry for Pakistan and China. This aligns with the research conducted by Darzi and Bhat [60], who examined the Pakistani fashion-wear business and investigated how customer satisfaction mediates the connection between brand image and customer retention. Their research revealed that satisfaction is a pivotal mediator affecting brand image's influence on customer retention in this particular business. Hence, the theoretical framework proposed by the current study offers fundamental support for our reported mediation effect. Our findings suggest that customer satisfaction mediates between customer trust and customer retention behavior.

Similarly, the current study findings show customer satisfaction mediates the relationship between cultural differences and customer retention behavior in the Bank for Pakistan and China. However, Gong and Yi [105] investigate the function of customer satisfaction as a mediator in cross-cultural service interactions. The theoretical approach proposed by Gong and Yi [105] offers conceptual validation for the mediation effect we have discovered. Our findings highlight the significance of customer satisfaction as a key mediator between cultural disparities and subsequent customer retention behavior. Our research provides empirical data supporting the theoretical position that cultural variations have a complex influence on customer retention, specifically via the lens of customer satisfaction. Therefore, our research adds to the ongoing discussion on how customer satisfaction mediates cultural characteristics and customer behaviors. This provides practical insights for banks that operate in culturally varied settings.

### 6.2 Moderating role of financial technology

The research provides strong evidence that financial technology positively moderates the relationship between customer satisfaction and customer retention behavior in China, but it is insignificant in Pakistan. These findings emphasize the growing importance of technical progress in influencing tactics for customer loyalty and retention [106].

According to the study's results, bank financial technology adoption is directly influenced by a bank's reputation, which is closely linked to customer satisfaction of a financial service provider in China. Customers' choices to defect are influenced by this as well, especially when it comes to controlling bank service lapses. As a stand-in for their total experience, clients in this situation look to a bank's financial technology as a critical signal for assessing the expected level of service [107]. Badzestau [108] explored that the significant changes in consumer behavior seen during the COVID-19 pandemic had far-reaching effects on a variety of service industries, including the financial services sector, in addition to retail marketplaces. For financial institutions, being able to provide efficient services has become a viable approach. Our findings argue that it is inevitable that bank clients will embrace efficient service quality, which is made possible by digital revolutions. Similarly, Jameaba [109] has stated that this transition is highlighted by consumers' greater knowledge of digital technology and their heightened expectations about competent service aspects.

### 6.3 Findings and discussion based on combine data: N = 593

On the other hand, the current study employed combined data: N = 593 for both China and Pakistan. Combined data analysis suggests that enhancing service quality improves customer satisfaction and customer retention behavior in Pakistani and Chinese banks. Akhtar et al. (2015) examined how service quality affects customer satisfaction in Pakistani banks and found that service quality was most essential [110]. This study verifies long-held beliefs regarding the link between customer satisfaction, loyalty, retention rates, and bank service. The Chinese banking business, Wang, and Zhang [111] demonstrated that better service boosts customer satisfaction and loyalty. Based on these results, banks in both countries should prioritize customer service to retain and attract consumers.

The literature on service quality and consumer behavior supports the idea that Chinese and Pakistani banks need service quality to retain and satisfy customers. Win [112]utilized this approach to study service quality and customer satisfaction in banking, supporting that service quality is crucial to consumer pleasure and loyalty. Thus, Chinese and Pakistani banks may improve service quality to improve customer happiness and retention.

The empirical evidence supports the hypothesis that customer trust significantly affects customer satisfaction and retention in the banking industry. Hidayat and Idrus [113] highlight the crucial role of trust and commitment in relationship marketing. The research conducted by Negassa and Japee [114] showed the positive impact of consumer trust in a service provider on perceived value, customer satisfaction, and loyalty. The significance of this connection is especially crucial within the banking industry since trust encompasses the level of faith that clients have in their financial institutions to demonstrate competence and fairness in their actions. Furthermore, within the realm of online banking and commerce, the significance of trust is heightened as a result of the absence of face-to-face engagement, hence making the building of trust a crucial obstacle. Al-Adwan, and Kokash [115] emphasized that trust plays a crucial role in predicting client retention in online contexts, as it helps to reduce the perceived risks connected with virtual transactions. These studies provide collective evidence that the cultivation of trust within the banking industry is crucial for improving customer satisfaction and guaranteeing customer retention. This highlights the strategic significance of trust as a fundamental element of relationship marketing and customer relationship management in both traditional and online banking environments.

The results of the study demonstrate that cultural difference has a significant effect on customer satisfaction [101]. However, these findings do not significantly affect customer retention behavior in the banking industries of Pakistan and China. These findings highlight the complex connection between cultural aspects and customer perceptions. This observation is consistent with other scholarly investigations that have emphasized the significant influence of cultural elements on the formation of people's views, beliefs, and actions. Pratesi, Hu [116] has shown that cultural factors such as individualism-collectivism and power distance substantially impact consumer decision-making processes and buying patterns. Therefore, although the research indicates that cultural disparities may not directly influence customer retention patterns within these banking settings, their significant impact on customer satisfaction necessitates that banks consider cultural subtleties in their service provision and communication approaches to improve overall customer experience and satisfaction levels. The results pertaining to the mediating role of customer satisfaction are in accordance with the findings of the present research, which performed distinct data analyses for samples from Pakistan and China.

The analysis of the combined data indicates that financial technology has a moderating effect on the connection between consumer satisfaction and customer retention behavior in the banking industry, which differs from previous research findings. Notably, distinct examinations reveal a considerable moderating impact in China, whereas no such effect is seen in Pakistan. However, a combined data analysis highlights the significant impact of financial technology on customer satisfaction and retention dynamics in the banking sector [117]. Hence, our findings argue that allocating resources toward financial technology is crucial for banks to leverage potential avenues for expansion, foster innovation, and sustain a competitive advantage within the market.

## 7 Conclusion

Our investigation sought to explore the impact of service quality, customer trust, and cultural differences on customer satisfaction within the banking industry. To scrutinize the distinct effects of service quality, customer trust, and cultural differences, we employed a diverse sample drawn from Pakistan and China. Additionally, our study aimed to delve into the mediating role of customer satisfaction and the moderating role of financial technology. The findings underscore the significance of these factors in influencing customer satisfaction, providing a comprehensive understanding of the complexities inherent in the contemporary banking landscape. Furthermore, our analysis extended beyond the examination of direct links, concentrating on the mediating role of customer satisfaction in the relationship between service quality, cultural differences, and customer retention behavior. This facet enhances existing knowledge by uncovering the underlying processes that interconnect these variables, presenting a more nuanced perspective on the factors contributing to client loyalty in the banking sector.

Moreover, considering its rapid evolution our study explored the moderating effect of financial technology on the link between customer satisfaction and retention behavior. This comprehensive approach contributes to a more thorough comprehension of the intricate dynamics in the intersection of customer satisfaction, technological advancements, and retention behavior in the banking industry. By considering this component, our study recognizes the transformational effect of financial technology improvements in the banking industry. This adds a modern perspective to the existing literature. It also emphasized the importance of considering technical factors while studying customer behavior and customer satisfaction in the contemporary banking industry. Our research significantly impacts the academic discussion on customer satisfaction and retention in the banking business and provides valuable insights for professionals in the field. Including cross-cultural factors enhances our results' relevance, acknowledging the worldwide scope of the banking sector. Future studies might investigate other contextual aspects and expand the area of analysis to new countries, therefore enhancing our comprehension of the complex nature of client interactions in the changing banking industry.

### 7.1 Implications

This study has significant consequences for both the academic and industrial sectors. Our investigation of the impact of service quality, customer trust, and cultural differences on customer satisfaction enhances the depth of knowledge in the current body of research. Our research improves the existing information by clarifying the complex interaction between cultural variations, service quality, and customer trust. This offers a detailed comprehension of how these elements jointly influence customer satisfaction.

Furthermore, our examination of the mediating role of customer satisfaction reveals a crucial connection between the quality of service, cultural disparities, and customer loyalty, supporting the crucial essential influence of customer trust on developing enduring customer relationships. Our research emphasizes the need for companies and financial institutes to prioritize establishing and preserving customer trust.

The current study investigates the impact of financial technology on the connection between customer satisfaction and retention behavior. Our results highlight the banking industry's changing nature and financial technology's increasing influence in altering customer retention behavior. It emphasizes adopting a human-centered approach when incorporating financial technology solutions. This suggests that effectively incorporating financial technology into customer relationship management requires a careful equilibrium between technology progress and human interactions to cultivate long-lasting client connections in the constantly changing financial services industry.

### 7.2 Study limitations and future study

The current study's investigation has uncovered several limitations. Using a random sampling process through an online survey can limit the generalizability of the results, even if the participants' characteristics roughly correspond to those of the Pakistani and Chinese populations. Moreover, the applicability of the research outside Pakistani and Chinese may be limited due to cultural, geographical, and economic constraints. The study mainly focuses on analyzing customer retention behavior; however, other factors like customer attitude, perceived behavior, and subjective norms may be incorporated into future studies. Therefore, future investigations might use longitudinal studies to analyze the chronological advancement of the mentioned elements.

## Funding disclosure

This research has been funded by the 10.13039/501100001809National Natural Science Foundation of China under fund number 72163018.

## Additional information

Supplementary content related to this article has been published online at https://doi.org/10.17632/y3s53svgvf.1.

## Ethical approval statement

Ethical approval for this research was obtained from the institutional Ethics Committee, Faculty of Management and Economics, Kunming University of Science and Technology under reference number 01214/2023. All participants provided informed consent before their inclusion in the study, and their confidentiality and anonymity were strictly maintained throughout the research process.

## Data availability statement

Data will be made available on request.

## CRediT authorship contribution statement

**Chai Zhengmeng:** Supervision, Resources, Project administration. **Muhammad Malik:** Writing – original draft, Software, Methodology, Formal analysis, Data curation, Conceptualization. **Muttahir Hussain:** Writing – review & editing, Validation, Data curation. **Salamat Hussain:** Visualization, Investigation.

## Declaration of competing interest

The authors declare the following financial interests/personal relationships which may be considered as potential competing interests:Chai Zhengmeng reports financial support was provided by 10.13039/501100001809National Natural Science Foundation of China. If there are other authors, they declare that they have no known competing financial interests or personal relationships that could have appeared to influence the work reported in this paper.
