# Supplementary material for: Exploring customer retention dynamics: A comparative investigation of factors affecting customer retention in the banking sector using mediation-moderation approach
Source: Heliyon. 2024 Aug 29;10(19):e36919. doi: 10.1016/j.heliyon.2024.e36919 (PMC11483330; doi:10.1016/j.heliyon.2024.e36919)
Supplement: Multimedia component 3 [file mmc3.docx]

**SURVEY QUESTIONNAIRE**

We are Ph.D. scholars of Kunming University of Science and Technology, as a mandatory part of the Ph.D. program we are conducting research on **Exploring Customer Retention Dynamics: A Comparative Investigation of Factors Affecting Customer Retention in the Banking Sector Using Mediation-Moderation Approach.**

Your information will be kept confidential and will not be used other than for research purposes. if you agree we need your input and your suggestions will be highly appreciated.

Thanks for your cooperation!

E-mail:

**Gender: Male Female**

**Age:** **18-25 Years 26-35 Years 36-50 Years**

**Status**:

**Student Employed Businessman**

**Education: Bachelors Masters PhD**

**Experience of Bank Account Holders 1-5 Years 6-10 Years 11-15 Years**

**Location/ country Pakistan China**

#### Indicate (✔) your level of agreement with the following statements about the “Customer Retention Behavior”.

**1. Service Quality**

| **S.No.** | **Measurement** | **Strongly**  **Disagree** | **Disagree** | **Neutral** | **Agree** | **Strongly**  **Agree** |
| --- | --- | --- | --- | --- | --- | --- |
| 1 | The financial institution has a clear and transparent service routine, which helps develop its reputation for being reliable. |  |  |  |  |  |
| 2 | The bank has established a rigorous method and system to effectively handle client data in a safe manner. |  |  |  |  |  |
| 3 | The Bank guarantees the precision of its comprehensive service method. |  |  |  |  |  |
| 4 | Service officers efficiently provide easy services, reducing any complex procedures for clients. |  |  |  |  |  |

**2. Customer Trust**

| **S.No.** | **Measurement** | **Strongly**  **Disagree** | **Disagree** | **Neutral** | **Agree** | **Strongly**  **Agree** |
| --- | --- | --- | --- | --- | --- | --- |
| 1 | The availability of my bank manager in difficult circumstances is constantly trustworthy. |  |  |  |  |  |
| 2 | The bank manager regularly makes beneficial suggestions to facilitate the development of my firm. |  |  |  |  |  |
| 3 | The advice given by my bank manager enhances my trust in my decision-making process. |  |  |  |  |  |
| 4 | I have confidence in my bank manager's competence to aggressively respond to and adjust to the changing financial needs of my firm. |  |  |  |  |  |

**3. Cultural difference**

| **S.No.** | **Measurement** | **Strongly**  **Disagree** | **Disagree** | **Neutral** | **Agree** | **Strongly**  **Agree** |
| --- | --- | --- | --- | --- | --- | --- |
| 1 | The cultural characteristics in Pakistan have a crucial impact on enhancing customer satisfaction and encouraging the long-term use of financial services. |  |  |  |  |  |
| 2 | The cultural backdrop has a vital role in improving customer satisfaction and promoting the long-term use of financial services. |  |  |  |  |  |
| 3 | The promotion of ethical and cultural values in financial operations has a substantial impact on customer decision-making in the banking industry. |  |  |  |  |  |
| 4 | The bank's strong engagement in charitable efforts and community initiatives has a positive impact on customer retention and devotion. |  |  |  |  |  |

**4. Customer Satisfaction**

| **S.No.** | **Measurement** | **Strongly**  **Disagree** | **Disagree** | **Neutral** | **Agree** | **Strongly**  **Agree** |
| --- | --- | --- | --- | --- | --- | --- |
| 1 | I made a wisely option in selecting this service provider. |  |  |  |  |  |
| 2 | The bank continuously fulfils my expectations by providing services of exceptional quality |  |  |  |  |  |
| 3 | Overall, my experience with this banking institution has been acceptable. |  |  |  |  |  |
| 4 | Overall, I feel satisfied with this bank |  |  |  |  |  |

**5. Financial technology**

| **S.No.** | **Measurement** | **Strongly**  **Disagree** | **Disagree** | **Neutral** | **Agree** | **Strongly**  **Agree** |
| --- | --- | --- | --- | --- | --- | --- |
| 1 | The effectiveness of Internet transactions enhances my trust in the bank. |  |  |  |  |  |
| 2 | I expect that the performance of Internet banking will be comparable to that of other technologies, such as telephone or TV banking. |  |  |  |  |  |
| 3 | I had a strong belief in the dependability of online banking, and it lived up to my expectations. |  |  |  |  |  |
| 4 | Utilizing financial technology enables me to conveniently reach my bank at any time, hence boosting flexibility in business hours. |  |  |  |  |  |

**6. Customer retention behavior**

| **S.No.** | **Measurement** | **Strongly**  **Disagree** | **Disagree** | **Neutral** | **Agree** | **Strongly**  **Agree** |
| --- | --- | --- | --- | --- | --- | --- |
| 1 | Customer feedback is systematically gathered on a regular schedule every week. |  |  |  |  |  |
| 2 | Personalized communications contribute to my perception of success, cultivating strong connections with the bank. |  |  |  |  |  |
| 3 | The bank fosters a culture that places client satisfaction as the highest priority. |  |  |  |  |  |
| 4 | The bank organizes regular client meetings to promote participation and communication. |  |  |  |  |  |

**Suggestions:**

**Thank you so much for your cooperation**
